# There’s more to death than life: Noncatalytic functions in kinase and pseudokinase signaling

**DOI:** 10.1016/j.jbc.2021.100705

**Published:** 2021-04-22

**Authors:** Peter D. Mace, James M. Murphy

**Affiliations:** 1 Biochemistry Department, School of Biomedical Sciences, University of Otago, Dunedin, New Zealand; 2 Inflammation Division, Walter and Eliza Hall Institute of Medical Research, Parkville, Victoria, Australia; 3 Department of Medical Biology, University of Melbourne, Parkville, Victoria, Australia

**Keywords:** pseudoenzyme, dead enzyme, nonenzyme, protein kinase, protein structure, BUBR1, BUB1 related-1, DSF, differential scanning fluorimetry, EGFR, epidermal growth factor receptor, HDX-MS, hydrogen-deuterium exchange mass spectrometry, HER, Receptor tyrosine-protein kinase erbB, ILK, integrin-linked kinase, IRAK, IL-1 receptor-associated kinase, JAK, Janus kinase, KSR, kinase suppressor of Ras, MEK1, MAPK/ERK kinase 1, MLKL, mixed lineage kinase domain-like, PABPs, poly(A)-binding proteins, PEAK, pseudopodium-enriched atypical kinase, PK-1, protein kinase-1, PsK, pseudokinase, RAF, rapidly accelerated fibrosarcoma kinase, RIPK, receptor interacting serine/threonine kinase, RKS1, resistance-related kinase 1, SgK, Sugen kinase, TRIB, Tribbles family pseudokinase, ULK4, Unc-51-like kinase 4

## Abstract

Protein kinases are present in all domains of life and play diverse roles in cellular signaling. Whereas the impact of substrate phosphorylation by protein kinases has long been appreciated, it is becoming increasingly clear that protein kinases also play other, noncatalytic, functions. Here, we review recent developments in understanding the noncatalytic functions of protein kinases. Many noncatalytic activities are best exemplified by protein kinases that are devoid of enzymatic activity altogether—known as pseudokinases. These dead proteins illustrate that, beyond conventional notions of kinase function, catalytic activity can be dispensable for biological function. Through key examples we illustrate diverse mechanisms of noncatalytic kinase activity: as allosteric modulators; protein-based switches; scaffolds for complex assembly; and as competitive inhibitors in signaling pathways. In common, these noncatalytic mechanisms exploit the nature of the protein kinase fold as a versatile protein–protein interaction module. Many examples are also intrinsically linked to the ability of the protein kinase to switch between multiple states, a function shared with catalytic protein kinases. Finally, we consider the contemporary landscape of small molecules to modulate noncatalytic functions of protein kinases, which, although challenging, has significant potential given the scope of noncatalytic protein kinase function in health and disease.

Protein kinases are quintessential signaling proteins. Their ability to posttranslationally modify amino acid side chains with a phosphoryl group underlies a broad swath of eukaryotic biology (1) and regulates protein activity in diverse pathways. In addition to catalyzing phosphoryl transfer, protein kinases also function in noncatalytic roles, interacting with other proteins and modifying their activity. A significant proportion of kinases even take noncatalytic function to the extreme—lacking phosphoryl-transfer activity completely—and are known as pseudokinases (2, 3, 4). Originally thought to be dead evolutionary remnants, pseudokinases have since been revealed to play remarkably diverse noncatalytic roles in biological pathways (5). Importantly, these zombie proteins provide a window into understanding the often unheralded, nonenzymatic functions that can be performed by their alive enzyme counterparts.

Catalytically competent protein kinases are diverse, but their shared enzymatic activity means that their protein folds are similar and core catalytic elements show little variation (Fig. 1*A*). The key elements for protein kinase catalysis are the ability to bind ATP, coordinate Mg^2+^, and catalyze phosphoryl transfer. These core elements generally consist of: a lysine residue within the VAIK motif in the N-terminal lobe, and a glycine rich loop (Gly-loop), which are key features for enabling ATP binding; an aspartate from the DFG-motif in the activation loop that coordinates magnesium alongside ATP; and an aspartate from the HRD loop contributed from the C-terminal lobe, which acts as a catalytic base during phosphoryl transfer (Fig. 1*B*; (2, 6)). Any, or multiple, of these elements can be lost in pseudokinases (7, 8, 9). Depending on what elements are lost, pseudokinases may be unable to bind nucleotides or magnesium (Class I), bind nucleotides but not cations (Class II), bind cations only (Class III), or bind both nucleotides and cations, but are still unable to carry out phosphoryl transfer (Class IV) (Fig. 1*C*) (9).

**Figure 1:**
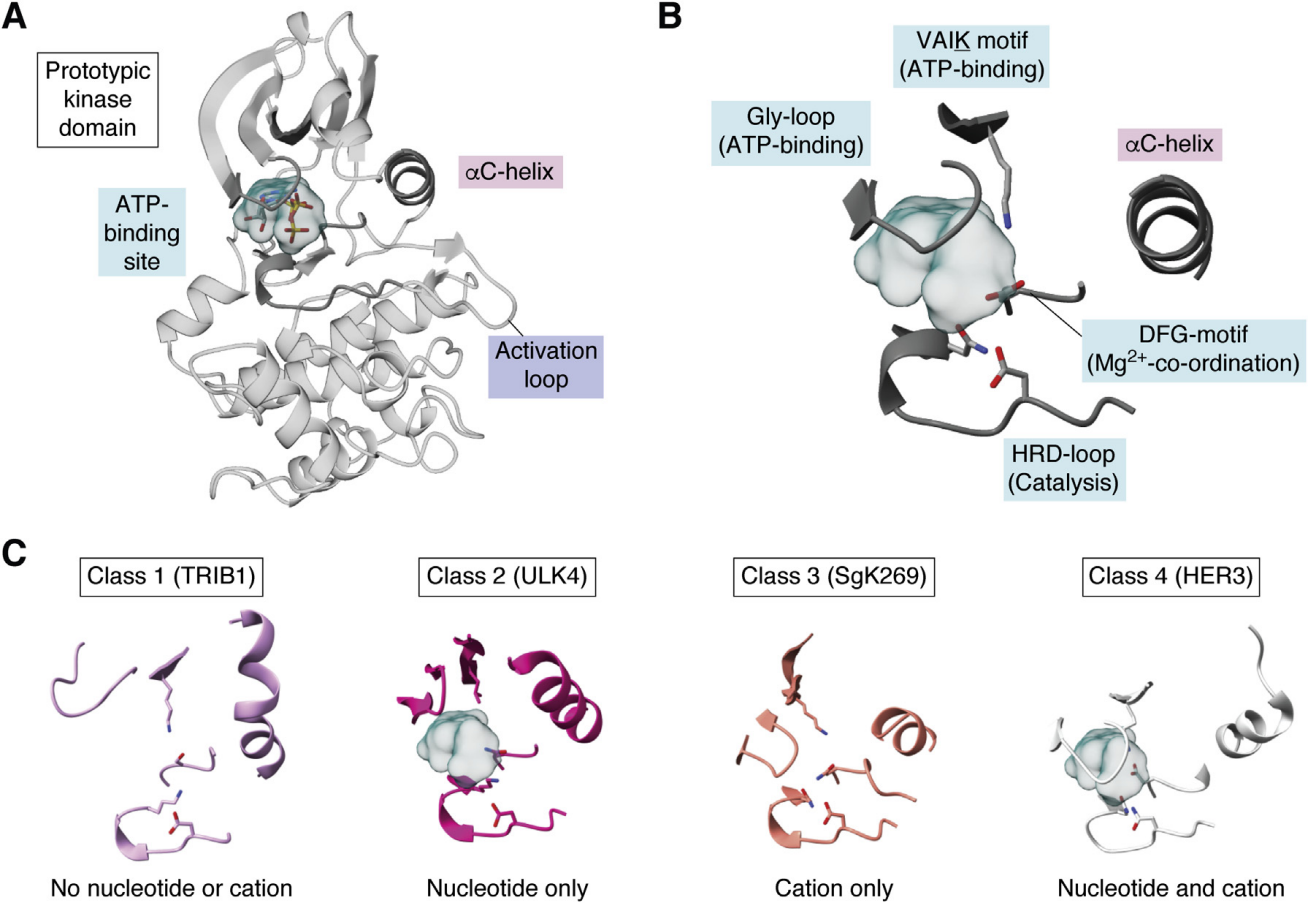
**Architecture kinases and pseudokinases.***A*, architecture of the prototypic protein kinase domain as demonstrated by PKA (PDB 1atp). *B*, close-up view of the PKA active site, with key catalytic features described in the text indicated. *C*, examples of the four classes of pseudokinase active site exemplified by TRIB1 (PDB 6dc0; (75)), ULK4 (PDB 6tsz; (81)), SgK269 (PDB 6bhc; (67)), and HER3 (PDB 3kex; (109)). For each, the Gly-loop, VAIK-motif, αC-helix, DFG-motif, and HRD catalytic loop are depicted, and pseudokinases that bind nucleotide (either without or with Mg^2+^; Classes 2/4) also have the nucleotide-binding site shown as a cyan surface.

Analyses of protein coding genes across archaea, bacteria, and eukaryotes have identified protein kinases, and pseudokinases, in all domains (8). This review focuses on the eukaryotic protein kinase fold, which is predicted to be present in low abundance in archaea and bacteria (10, 11), although our understanding of protein kinases more broadly in prokaryotes is still emerging (12, 13). The human proteome has long been known to contain approximately 550 protein kinases, of which approximately 10% are pseudokinases. This proportion of noncatalytic kinases is generally retained across vertebrates, with approximately ∼10% of protein kinomes designated as pseudokinases (2, 14). More broadly, some eukaryotic species have expanded pseudokinase complements. For instance, the kinomes of plants frequently comprise up to ∼17% pseudokinases, and approximately half of kinase-like proteins in selected protists (*Toxoplasma gondii* and *Giardia lamblia*) lack essential catalytic residues (8). Such pseudokinase expansion is frequently concentrated in specific classes of kinases. For example, plants have undergone a massive expansion of pseudokinases, likely due to their important role in innate immunity (15). The broad scale of current analyses means that most pseudokinase classification is sequence-based rather than experimentally verified. While computational approaches have been enlightening, several pertinent examples demonstrate the need to couple with experimental characterization. For instance, kinases with seemingly degraded catalytic sequences can nonetheless retain the ability to phosphorylate proteins or other specific biomolecules (16, 17, 18, 19) or can carry out completely unanticipated catalytic functions distinct from phosphoryl transfer (20, 21, 22, 23). Nonetheless, coupled bioinformatic and experimental approaches will be essential for continued insight into the conserved and important noncatalytic roles played by kinases throughout evolution.

Pseudokinases have led the emerging realization that catalytically inactive enzymes (pseudoenzymes) play roles in almost all facets of biology (24). Across protein families and kingdoms of life, proteins that have an enzymatic fold but lack catalytic activity regulate biological processes through a number of different mechanisms (24, 25, 26). Pseudoenzymes include pseudo-phosphatases, pseudoproteases, and pseudoGTPases, among others. Broadly speaking, pseudoenzymes function as: allosteric activators, competitive inhibitors, scaffolds for assembly of protein complexes, or as protein switches (Fig. 2; (25)). Examples in each of these categories show that regulatory features and interaction surfaces evolved for catalytic proteins can be retained or repurposed toward alternative biological function in noncatalytic versions of the same protein or eschewed completely to evolve new activity. Thus, while pseudoenzymes are noncatalytic, this does not mean they are nonfunctional. It is also important to note that pseudoenzymes are not the same as pseudogenes. Pseudogenes refer to incomplete DNA sequences lacking regulatory elements, whereas pseudoenzymes are translated proteins encoded by functional genes.

**Figure 2:**
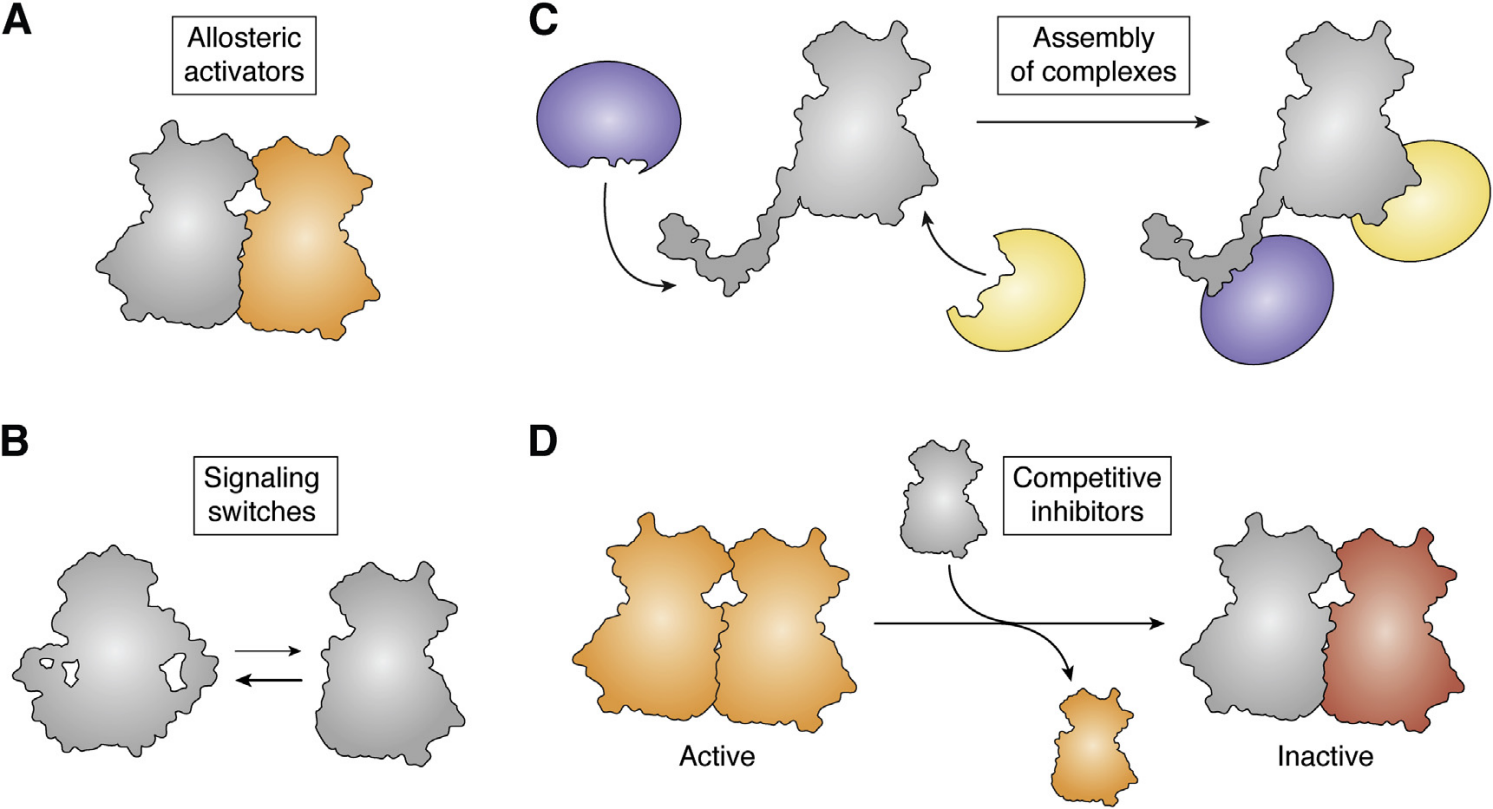
**Modes of noncatalytic kinase activities.** Cartoon depiction of the four modes of noncatalytic kinase function: (*A*) allosteric activators, (*B*) signaling switches, (*C*) assembly of complexes, and (*D*) competitive inhibitors. In each case, kinase domains that play noncatalytic roles are depicted in *gray*.

Here, we focus on recent illustrative examples for understanding noncatalytic functions of protein kinases at the molecular level. Because they are by definition noncatalytic, pseudokinases provide many of the clearest examples of noncatalytic regulation and function across each of the known categories of pseudoenzymes. Noncatalytic kinase function is particularly linked to function as protein switches, because the core architecture of the kinase domain encodes the ability to switch between on- and off-states. Many pseudokinases have retained the ability to switch between different conformations even though they lack core catalytic elements. This means that some pseudokinases can function as switches to regulate activity, but simultaneously as scaffolds, inhibitors, or activators. Thus, when freed from the constraints of retaining enzymatic activity, protein kinases can elaborate on functions beyond catalysis or develop completely novel roles as dead enzymes. Accordingly, dead enzymes offer exemplars of additional, often unrecognized noncatalytic functions that might be performed by conventional, alive enzymes—in keeping with the idea that, at least in the case of enzymes, there is more to death than life.

## Allosteric activation

One of the best-characterized functions of pseudokinases is their modulation of cognate kinases, by either promoting or attenuating catalytic activity of their binding partners. Because pseudokinases have often arisen from gene duplication events, they frequently function within the same pathway as their cognate kinase partners owing to common expression patterns and subcellular localization, as noted previously (26, 27, 28). Such duplications bring enormous liberty; because of the functional redundancy that arises within a pathway through duplication, there is no necessity to maintain active site catalytic residues or geometry to mediate phosphoryl transfer. One of the most striking examples is in the Janus Kinase (JAK) family, where a pseudokinase domain (termed JH2) occurs in tandem, N-terminal to the catalytically active tyrosine kinase domain (termed JH1) and attenuates its catalytic activity, likely in *trans* within receptor-scaffolded dimers (29, 30, 31). While the mechanism is still debated (31), this function was clearly revealed by the discovery of activating pseudokinase domain mutations (32), which promote JAK2 signaling and induce hematopoietic malignancies. Accordingly, from duplications of their kinase ancestors, pseudokinases can evolve pseudoactive sites that do not bind nucleotide, diminish their activation loops, and adopt conformations discordant with catalytic activity. Any of these modifications enable function as protein interaction domains that regulate activities of their cognate kinase partners allosterically.

**Figure 3:**
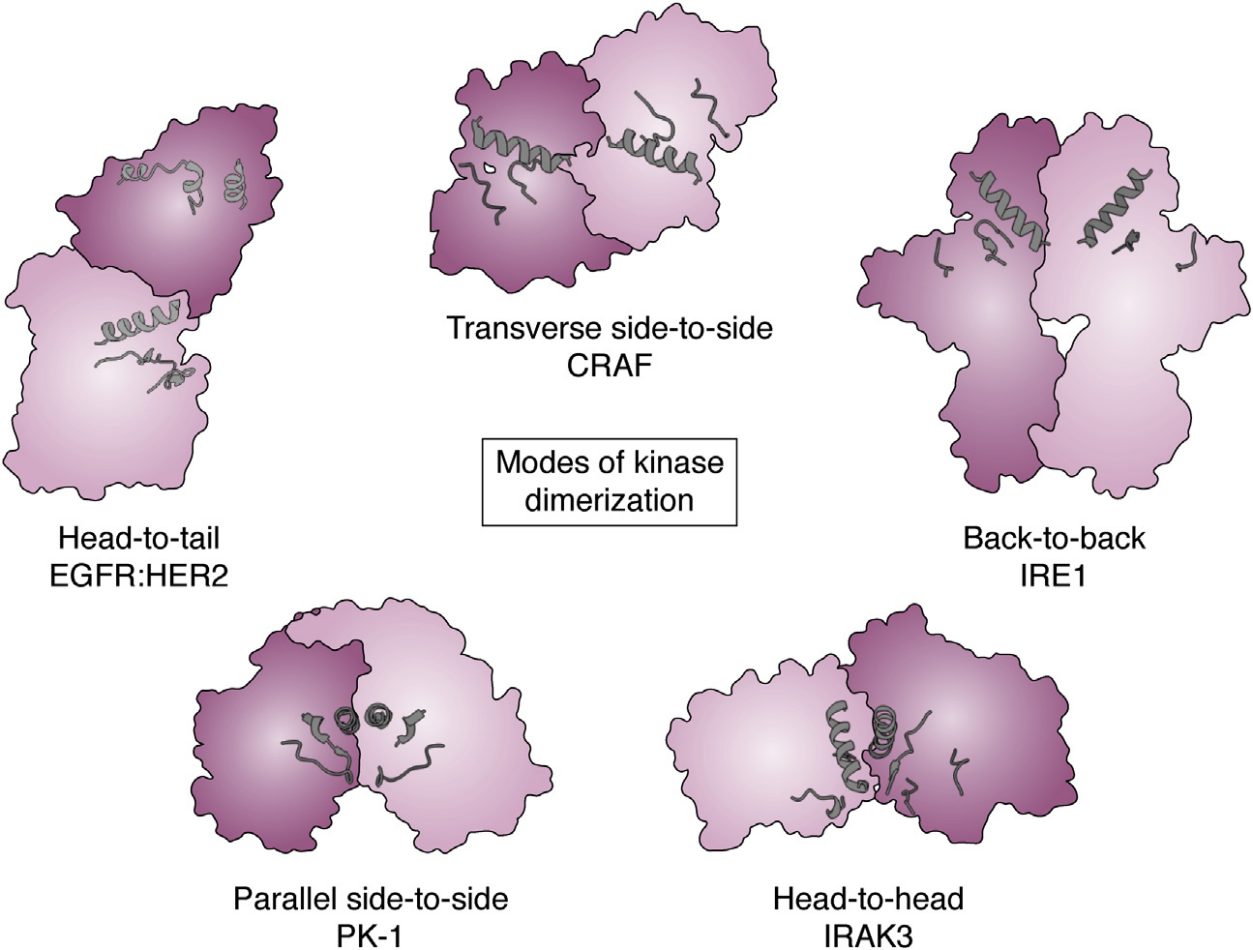
**Modes of kinase dimerization.** Examples of the five different modes of kinase dimerization described in the text. Structures displayed are EGFR:HER3 (PDB 4riw; (38)), CRAF (PDB 3omv; (41)), IRE1 (PDB 2rio; (36)), PK-1 (PDB 6vvg; (34)), and IRAK3 (PDB 6ruu; (39)), with the αC-helix and activation loop depicted as *ribbons* in each.

*Via* intermolecular interactions, kinases and pseudokinases are able to modulate the position of the key regulatory element, the αC helix within the N-lobe of the kinase fold, to promote active or inactive conformations of the catalytically active partner kinase. Several distinct modes of dimerization have been reported to influence the position of αC helix, which have been illuminated by detailed structural studies, and highlight the versatility of the kinase fold as a protein interaction domain (Fig. 3; (33, 34, 35)). Many of the different regulatory binding modes are illustrated by pseudokinase domain binding to a cognate kinase or pseudokinase domain, including: back-to-back (as observed for Ire1 and RNase L homodimers (36, 37), head-to-tail (as observed for EGFR family proteins, such as HER3 pseudokinase:EGFR kinase (38)), head-to-head (as found for IRAK3 homodimers and proposed for IRAK3 pseudokinase:IRAK4 kinase pairs (39)), and antiparallel side-to-side (exemplified for RAF:RAF kinase dimers and KSR pseudokinase:RAF kinase heterodimers (40, 41, 42)) modes. These studies raise the possibility that protein kinases may exert noncatalytic regulatory roles on other kinases, similar to those exerted by pseudokinases, as recently proposed for the parallel side-to-side mode of homodimerization reported for the granuloviral PK-1 kinase (34). While not yet observed among pseudokinase:kinase pairs, this binding mode couples dimerization with the αC helix occupying a position synonymous with catalytic activity.

Furthermore, while currently poorly understood, some pseudokinases have been reported to allosterically regulate the activities of nonkinase enzymes, as proposed for VRK3 pseudokinase binding to, and activation of, the VHR phosphatase (43, 44). Overall, these findings illustrate the breadth of noncatalytic allosteric functions that can be mediated by pseudokinase domains and suggest these may be underappreciated functions of protein kinases more generally. Deducing the precise nature of these noncatalytic allosteric functions of conventional protein kinases remains a major challenge. Such studies will rely on elegant chemical biology and catalytically dead knockin approaches, rather than gene deletion or knockdown, to reveal functions beyond phosphoryl transfer.

## Kinases and pseudokinases as molecular switches

Over the past 30 years, crystal structures of kinase and pseudokinase domains have captured the N- and C-lobes and the regulatory elements, the αC helix and activation loop, and structural pillars of hydrophobic networks (termed spines) in a continuum of conformations, illustrating their intrinsic dynamicity (45, 46, 47). In the case of conventional, active kinases, this flexibility has been associated with regulation of catalytic activity. Basally, the apoenzyme is proposed to exist in a catalytically uncommitted state until ATP binding, which galvanizes the protein’s internal hydrophobic networks and poises the kinase for catalysis. Allosteric effectors and oligomerization are known to modulate adoption of a catalytically active conformation signified by an intact regulatory (R)-spine and αC helix Glu engaged in a salt bridge with the β3-strand Lys (45). However, what if, more broadly, the range of conformations accessible by kinase and pseudokinase domains might reflect their propensity to serve as molecular switches? Recent studies have revealed that beyond the catalytic functions of kinases, both they and pseudokinases serve important signaling functions *via* protein–protein interactions. Consequently, an attractive hypothesis is that the propensity for these interactions could be governed by the conformation of the kinase or pseudokinase, and additionally, these conformations might be regulated by binding partners or posttranslational modifications.

**Figure 4:**
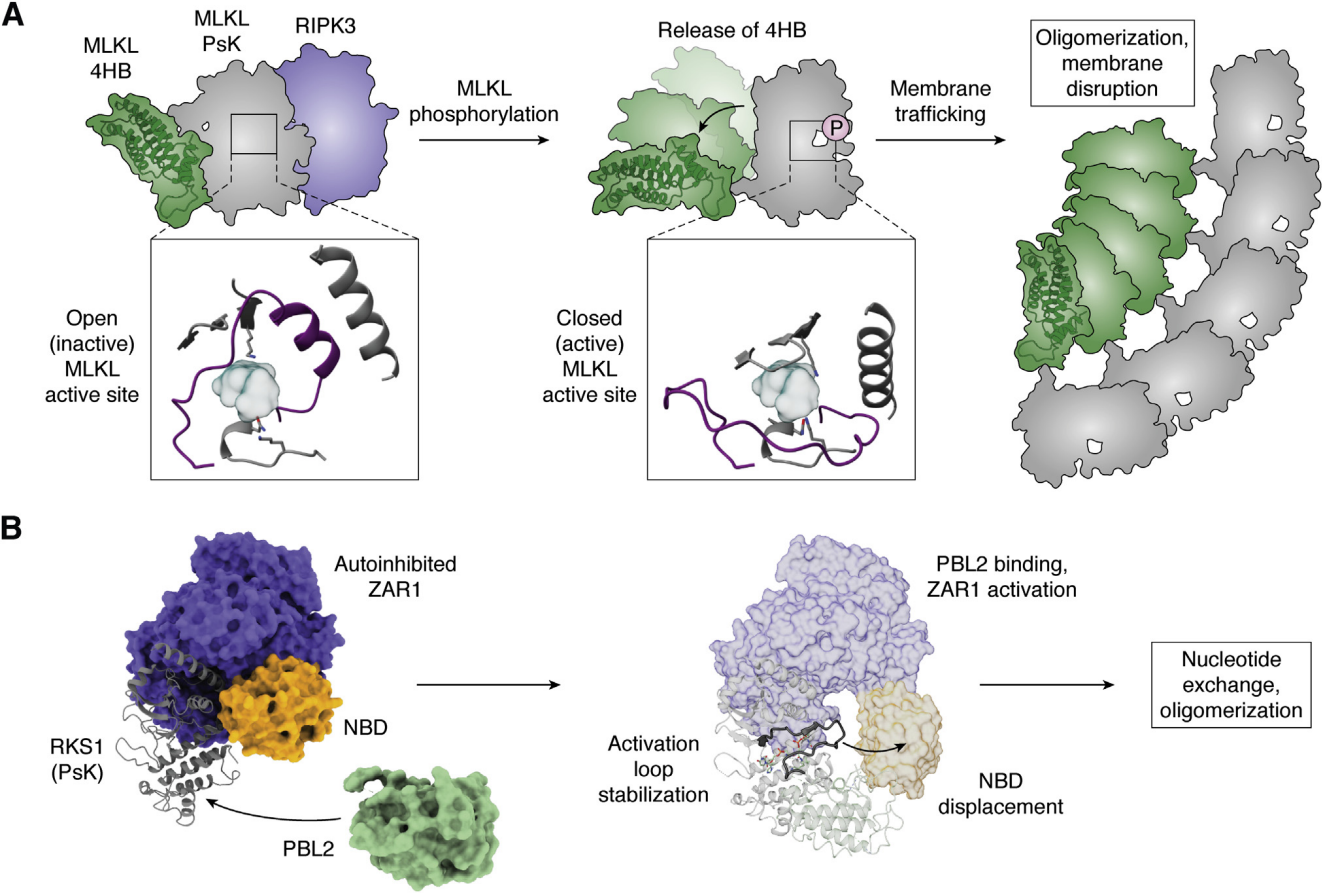
**Examples of noncatalytic kinase switching.***A*, schematic of the switch occurring upon MLKL activation. The inactive conformation (*left*) displays an intimate interaction between the pseudokinase domain and 4HB (killer) domain and an open active site and readily interacts with RIPK3. Activation of RIPK3 promotes phosphorylation of the activation loop, release of the 4HB, membrane trafficking, and permeabilization. *Inset* views of the MLKL active site are derived from a model of inactive human MLKL (PDB 4mwi; (57)) with the activation loop (*purple*) computationally modelled (as reported in (59)), and the active form from PDB 7jxu (59). *B*, summary of the activation mechanism of ZAR1. Uridylated PBL2 (*green*) binds to the RKS1 pseudokinase (*gray*) in such a manner that stabilizes the activation loop. Stabilization of the activation loop, in conjunction with the orientation of RKS1-ZAR1 binding displaces the ZAR nucleotide-binding domain (NBD; *yellow*), promoting nucleotide exchange and oligomerization. The composite illustration is derived from PDBs 6j5w, 6j5v and 6j5u (61).

The concept of the kinase fold being employed by nature as a molecular switch is best illustrated by the Mixed Lineage Kinase domain-Like (MLKL) pseudokinase. Unlike conventional kinases, it functions solely as a protein interaction domain, and thus interpretation of conformational effects is not confounded by an additional catalytic activity function exerted by active kinases. MLKL is the terminal effector in the necroptosis cell death pathway, which is a lytic cell death modality that, unlike the cousin pathway apoptosis, does not rely on the proteolytic functions of Caspases (reviewed in (48)). Instead, necroptosis arises following an insult, such as inflammatory death receptor signaling or activation of innate pathogen sensors, which leads to oligomerization and activation of the receptor-interacting protein kinase-3 (RIPK3) effector kinase by autophosphorylation (reviewed in (49)). Activated RIPK3 can then phosphorylate its substrate, MLKL, on the activation loop of its pseudokinase domain, to induce a conformational change that leads to exposure of the killer N-terminal four-helix bundle domain, MLKL oligomerization, and translocation to the plasma membrane (Fig. 4*A*; (50, 51, 52, 53, 54, 55)). At the membrane, MLKL accumulates into hotspots that permeabilize the lipid bilayer to cause cell lysis, death, and release of proinflammatory molecules (51, 56).

The idea that MLKL can function as a molecular switch has evolved through a series of detailed biochemical and structural studies. First and foremost, MLKL binds ATP, albeit in the absence of the conventional catalytic cofactor, Mg^2+^ (9, 55, 57). Because MLKL is noncatalytic, this raised the prospect that ATP binding may serve as a barometer to gauge its propensity to undergo conformational switching. Indeed, in the case of mouse MLKL, introduction of disruptive mutations in the pseudoactive site was sufficient to trigger its killer function (55). The proximity of this switch to the MLKL activation loop residues phosphorylated by RIPK3 provided a direct link between pseudoactive site integrity and activation loop phosphorylation (Fig. 4*A*). Together, these act as cues for conformational transitions that disable the suppressor function of the pseudokinase domain and promote the killer function of the N-terminal 4HB domain. While ATP binding does not appear to serve a physiological function in dictating MLKL activation, it does raise the question whether the ATP-binding site could be targeted pharmacologically to block conformational transitions and prevent errant MLKL activation (54, 58).

Structural interconversion prompted by MLKL pseudokinase domain activation loop phosphorylation by the upstream regulator, RIPK3, was proposed owing to the diversity of pseudokinase domain conformations observed in crystal structures (50, 59). Mouse MLKL adopts an open conformation with a disassembled R-spine, no β3 Lys:αC Glu salt bridge, and the activation loop forming an unusual helix (55). In contrast, human MLKL adopted a more active-like conformation with an intact R-spine and β3 Lys:αC Glu salt bridge and an unstructured activation loop (57). These distinct conformations were proposed to represent two phases of a molecular switch, which is supported by recent structures of the human MLKL pseudokinase domain captured in these two conformations in complexes with synthetic protein ligands termed monobodies (59). Coupled with earlier biochemical and structural studies, these structures are consistent with the open form as the conformer bound by RIPK3 prior to a stimulus provoking RIPK3 activation and MLKL phosphorylation; and the closed form representing the activated form of MLKL, whose transition to the closed conformer is promoted by phosphorylation and coupled to dissociation from RIPK3 (Fig. 4*A*, inset). These data implicate the noncatalytic binding of RIPK3 to MLKL as a regulator of MLKL conformation and thus whether MLKL can convert from a dormant to killer form. Reciprocally, precisely how the binding of MLKL, an intrinsically noncatalytic protein, to RIPK3 impacts RIPK3 structure, activation, and catalytic activity is currently an open question, whose answer relies on the future availability of additional structures beyond the mouse RIPK3 kinase domain (60).

The widespread retention of activation loops among pseudokinases raises the prospect that modulation of activation loop conformation, such as by phosphorylation or regulatory protein binding, may serve more broadly to toggle conformations to regulate noncatalytic functions. While MLKL illustrates the role of activation loop phosphorylation in conformational toggling (50, 59), the regulation of activation loop conformation by binding to a partner protein has been reported within the plant innate immunity complex between the ZAR1 Nod-like receptor protein, the RKS1 pseudokinase, and the PBL2 kinase (61). Pathogen uridylation of PBL2 leads to its function as a host detector of infection, whereby uridylated PBL2 binds to RKS1 within the ZAR1:RKS1 complex to induce an immune response (Fig. 4*B*). By binding to RKS1 and rigidifying the activation loop, PBL2 is thought to induce a structural change in which the nucleotide binding domain (NBD) of ZAR1 is displaced, enabling it to bind ATP to toggle to its “on” state as a host defense effector (61). Further knowledge of the prevalence of molecular switch mechanisms in nature awaits detailed studies of further pseudokinases. A major challenge with the study of pseudokinases is that they are not a family *per se*, but rather the zombie cousins of conventional kinases—renegade proteins integrated within the family tree, but walking down a different path. As such, understanding their underlying regulation, and whether they function as molecular switches, will rely on detailed structural studies of individual proteins, akin to those performed on MLKL and RKS1.

## Protein kinases as signaling scaffolds

By definition, protein kinases associate with other proteins to perform their function, meaning that the protein kinase fold is used throughout evolution to mediate protein–protein interactions. Multiple interaction surfaces of the kinase domain can interact with substrates, regulators, or binding partners, and such diversity of binding modes underlies one of the most common mechanisms of noncatalytic kinase function—complex scaffolding. Scaffolding is performed by both catalytically active and pseudokinase proteins alike and can exploit the ability of kinases to switch between “on” and “off” states inherent to the protein kinase fold.

As described above, dimerization of kinases for allosteric activation is a relatively common noncatalytic protein kinase function. In addition to direct allosteric activation, kinase dimerization can also scaffold the formation of larger signaling complexes. A very pertinent example is the multidomain, pseudokinase domain-containing protein Kinase Suppressor of Ras (KSR). The KSR pseudokinase domain can dimerize with Raf kinases to allosterically activate them (42, 62) and interact with MEK kinases (63), which can allosterically impact their susceptibility to small-molecule inhibitors (discussed below). However, KSR also has domains capable of binding to various players within the RAF-MEK-ERK cascade to regulate flux through the pathway, namely: a domain that specifically interacts with the N-terminal domain of RAF proteins; peptidic motifs that can be bound by the terminal MAP kinase of the cascade ERK to provide feedback (64); and docking sites for 14-3-3 proteins and phosphatases. As such, the KSR pseudokinase domain is one of several protein–protein interaction regions within a multidomain protein, allowing it to effectively recruit multiple relevant partners to facilitate a cohesive signaling cascade.

**Figure 5:**
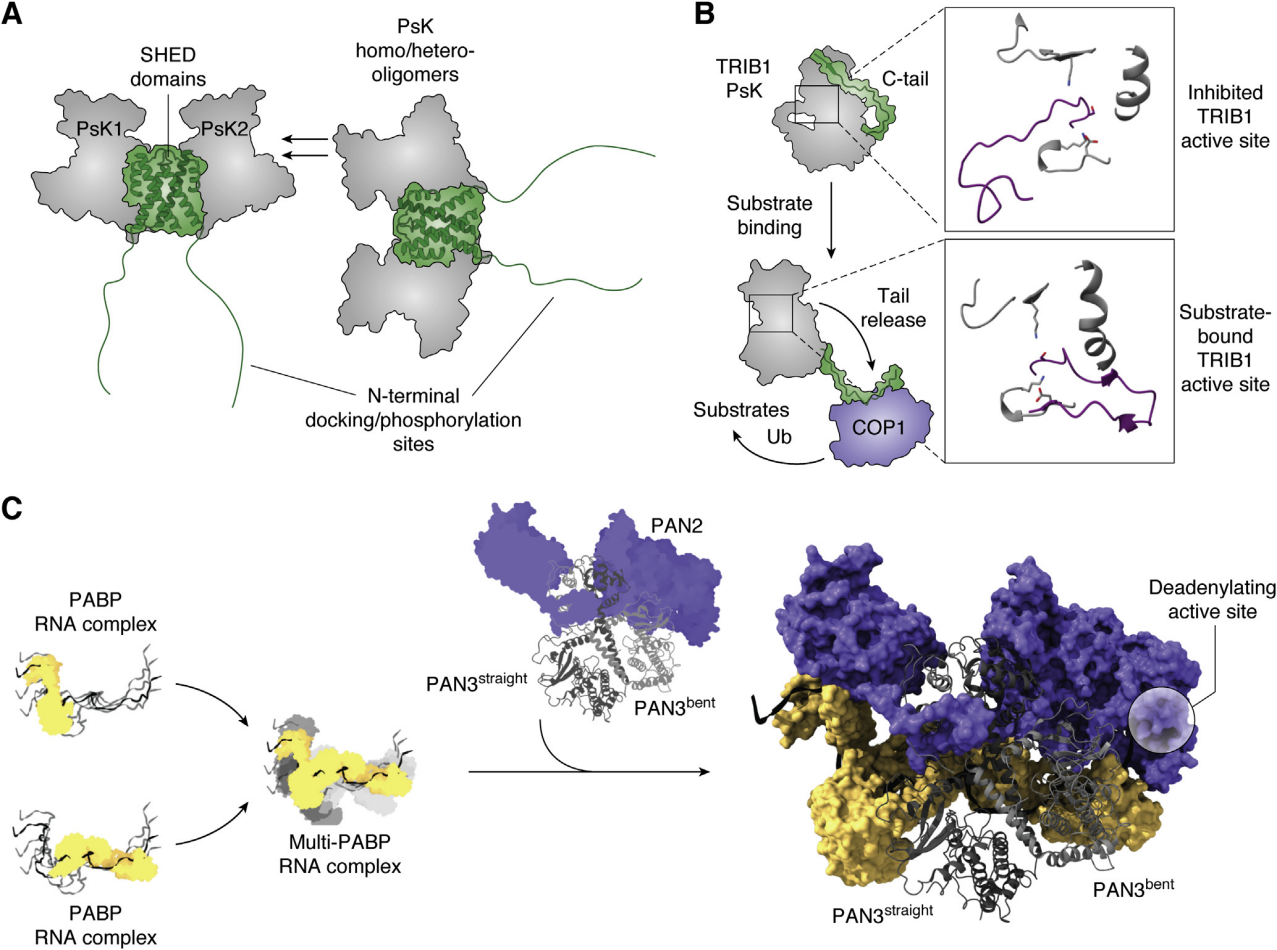
**Scaffolding roles of noncatalytic kinases.***A*, illustration of pseudokinase homo- and hetero-oligomerization by the PEAK family pseudokinases, derived from PDB 5ve6 (66). A tight interaction between SHED domains (*green*) brings together PEAK pseudokinase domains and also exposes another site located on the pseudokinase C-terminal lobe for weaker homo- or hetero-oligomerization. Overall this leads to clustering and brings together multiple docking sites located at the N-terminal regions of each protein. *B*, illustration of the activation mechanism of TRIB pseudokinases by substrate binding. In the inactive state, the COP1-binding motif of TRIB1 (*green*) is sequestered by the back side of the ɑC-helix. Substrate binding promotes a conformational change that rearranges the activation loop (*inset*; *purple*) and promotes release of the C-terminal COP1 binding site and COP1 binding. Active site depictions show the same elements as Figure 1, with inactive and active states derived from PDBs 5cem and 6dco respectively (75, 78). The disordered activation loop is computationally modeled for 5cem (as described (75)). *C*, illustration of the central role of the PAN3 pseudokinase domains in poly(A) recognition. PABP proteins individually recognize poly(A) sites, but maximum complex assembly is achieved when multiple PABPs are present. This complex is recognized by the Pan2/3 complex, with PAN3 bridging interactions between PAN2 and multiple PABP proteins. The structure on the *right* is derived from PDB 6r5k (92).

In a similar vein to the classical KSR scaffold, the PEAK proteins are a family of large multidomain scaffolding proteins containing a pseudokinase domain as a critical feature (65). The three family members (SgK269, SgK223, and PEAK3) all play roles as signaling hubs for different cellular processes, but particularly related to cytoskeletal and adhesion signaling. In each protein an array of docking sites for SH2 and SH3 domains and additional regulatory phosphorylation sites are found within the N-terminal portion and a pseudokinase domain toward the C-terminus (Fig. 5*A*). Crucially, the PEAK pseudokinase domains all harbor a bundle of helices that are central to a unique mode of dimerization. The N- and C-terminal helices have been termed the “SHED domain” and form extensive interactions with one another and leave the pseudokinase domain decorating the outside of the dimer (66). Crystal structures of the pseudokinase domains of SgK269 and SgK223 have shown very similar dimeric arrangements, and sequence conservation in PEAK3 suggests that it will also dimerize in a similar manner (66, 67, 68, 69). The accessibility of the pseudokinase on the outside of the dimer allows it to partake in weaker pseudokinase–pseudokinase interactions, thus building the possibility for both homotypic and heterotypic interactions (Fig. 5*A*). Much is still to be determined about the interplay between these pseudokinase oligomeric states and interactions mediated through the N-terminus, although PEAK dimerization appears to be key for their phosphorylation by Src family kinases and subsequent binding of adaptor proteins (CrkII, p130Cas; (69)). Given the roles of PEAK pseudokinases in cell motility and cancer, it will be an interesting to see if small molecules can effectively antagonize their function either directly or through targeted degradation—many of the demonstrably druggable pseudokinases exhibit nucleotide binding activity, whereas the PEAK family does not.

Another recently characterized mechanism of scaffolding by a pseudokinase domain is that of BUBR1 (BUB1 related-1), which is essential for regulation of the spindle-assembly checkpoint during mitosis. BUBR1 provides fertile ground for investigating the link between pseudokinase function and scaffolding by virtue of the fact that the BUBR1 pseudokinase, which possesses docking sites outside of the pseudokinase domain, and the partner catalytic kinase BUB1, have diverged through evolution. Moreover, there are divergent reports of nucleotide binding and activity by the *Drosophila* protein, compared with human BUBR1 (9, 70). Coevolution analyses suggest that a phosphorylation-dependent phosphatase docking site (known as a KARD motif) has been positively selected in conjunction with the BUBR1 pseudokinase domains (71). Mutational studies revealed that residues within the pseudoactive site of the BUBR1 pseudokinase domain, but not catalysis itself, are essential to the ability of BUBR1 to regulate KARD phosphorylation and scaffold assembly (71). Phosphorylation of the KARD motif in BUBR1 was essential as part of a phosphorylation relay, because it then facilitates scaffolding of an active complex with PP2A. The posited model proposes that an intramolecular interaction between the pseudokinase and KARD motifs promotes phosphorylation, potentially by stabilizing the KARD region (71). Such a model will be an intriguing to examine further, given the key role of BUBR1 in checkpoint silencing and mitotic exit. More generally, the BUBR1 pseudokinase also illustrates the clear utility of the kinase domain as a template to evolve diversity of protein–protein scaffolding interactions.

Beyond phosphorylation-related protein complexes, pseudokinases also act as scaffolds to bring together more disparate protein folds. The Tribbles family of pseudokinases (TRIB1–3 and the more distal SgK495) are a key example, bringing diverse proteins together to facilitate ubiquitination (72). The Tribbles family shares a common domain structure of a central pseudokinase domain flanked by N- and C-terminal extensions. In general, the pseudokinase domain is responsible for binding to substrates, while the C-terminal tail contains a motif that tethers Tribbles to the COP1 ubiquitin ligase. Thus, Tribbles proteins effectively function as substrate adaptors for ubiquitination and degradation. Tribbles proteins have overlapping and distinct substrates. For instance, TRIB1 and TRIB2 bind to C/EBP transcription factors to regulate blood cell development and leukemia (73, 74, 75). In contrast, TRIB3 is unable to bind C/EBPs, but is reported to regulate metabolism *via* engagement of acetyl-CoA carboxylase and AKT (76, 77). While TRIB3–acetyl-CoA carboxylase regulation seems to be analogous to TRIB1/2–C/EBP—through ubiquitination and degradation (76)—the mechanism of AKT regulation and structural basis for either interaction remains unclear. Structural knowledge of Tribbles pseudokinases is currently derived from TRIB1 and SgK495 (75, 78, 79). Notably the TRIB1 pseudokinase domain can bind its own COP1-binding motif, in a manner that prevents COP1 binding—effectively providing an autoinhibited state (78). To bind a specific degron in C/EBP proteins, TRIB1 undergoes a change in conformation from the autoinhibited state, thus substrate binding and recruitment to COP1 are allosterically linked (Fig. 5*B*; (75)). Most intriguing from the evolutionary perspective is that this mechanism centers on residues that have replaced the “DFG” motif of Tribbles pseudokinases—a Ser-Leu-Glu motif in TRIB1. It appears that Tribbles have not only lost residues required to coordinate magnesium for catalysis, but have done so by adopting alternatives that still enable a switching function quite similar to conventional kinases, which lends itself to a switchable scaffolding function.

Where TRIB1 appears to have eschewed the ability to bind ATP, this is not necessarily universal among scaffolding pseudokinases. There are reports that TRIB2 and TRIB3 retain some weak nucleotide binding and activity (80), and the ULK4 pseudokinase, which putatively functions as a protein scaffold, can bind ATP (9, 81, 82). The ULK4-ATP-binding mechanism is quite unconventional, whereby an alternative lysine residue compensates for the lack of an intact VAIK motif (81). Nonetheless ATP binding appears to be robust and Mg^2+^-independent (Fig. 1*C*). The unusual active site architecture is strongly correlated with an unusual activation loop conformation. How this pertains to binding of partner proteins at the centrosome and within microtubules remains to be determined. However, given the lessons from other pseudokinases, it is plausible that the specific divergent regions with the pseudokinase domain will be correlated with the functional mechanism. The ability to bind ATP appears to be crucial for the ability of the pseudokinase domain-containing protein, integrin-linked kinase (ILK), to modulate cell migration and matrix remodeling (83, 84). Binding of ATP by ILK is Mg^2+^-dependent (9) and stabilizes the pseudokinase domain such that it can more effectively mediate interaction between the focal adhesion adaptor proteins PINCH and Parvin in the process of cellular force generation (85, 86). Dependence of ILK function on the active site again raises the possibility for small-molecule antagonists that modulate scaffolding function.

Scaffolding of interactions by pseudokinases is not limited to complexes containing proteins. Detailed structural and biochemical analyses have elegantly demonstrated how the pseudokinase domain of Pan3 nucleates specific recognition and cleavage of mRNA poly(A)-tails. Pan3 contains a pseudokinase domain that forms asymmetric dimers (Fig. 5*C*; (87, 88, 89, 90). The deadenylating component of the Pan2/Pan3 complex is contained within the Pan2 partner (91), but how the complex specifically recognizes extended poly(A) tails and trimmed to a specific length was unclear until recently. It is now apparent that the pseudokinase domains of Pan3 act as an organizing hub, central to the organization of Pan2/3 relative to poly(A) tails and poly(A)-binding proteins (PABPs) (Fig. 5*C*; (92)). PABPs exist in cells at high concentration and bind poly(A) tails with high affinity. Pan2/3 engage most effectively with poly(A) tails bound by two or more PABPs, providing an initial recognition of length. Within the asymmetric pseudokinase dimer, the “bent” Pan3 pseudokinase mediates an intimate interaction with Pan2 while the “straight” conformer forms a large interaction surface with the proximal PABP protein. This arrangement clamps the polyadenylation machinery to one end of the RNA, while stabilizing interactions at the distal end with a second PABP molecule (Fig. 5*C*). This allows further interactions with PAN2 with the distal end of the RNA. Thus, the asymmetric Pan3 pseudokinase domains act as an organizing center, allowing for the Pan2/3 complex to specifically recognize poly(A) complexes of sufficient length.

## Kinases/pseudokinases as competitors in signal transduction

**Figure 6:**
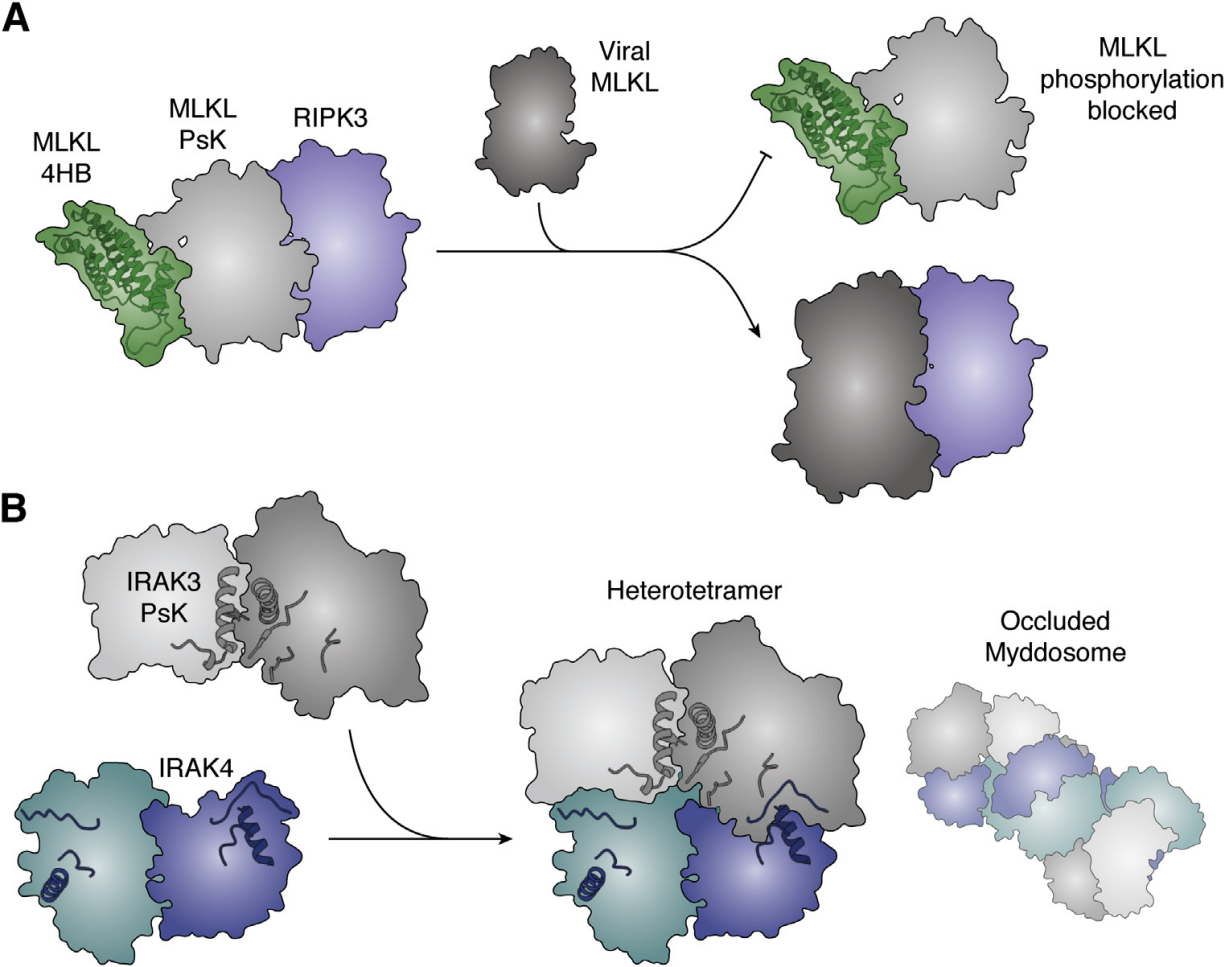
**Noncatalytic kinases as mediators and targets of inhibitors.***A*, illustration of the mechanism of viral MLKLs (*dark gray*), which compete with endogenous MLKL for binding to RIPK3 (*blue*). *B*, proposed interaction of IRAK3 pseudokinase homodimers binding to IRAK4 homodimers in an appropriate manner to suppress IRAK4 activity and potentially impede the assembly of higher-order IRAK4 activation at the Myddosome. Models derived from PDBs 6RUU and 4U97, as described in reference (39). Activation loops and αC-helices of IRAK3 and IRAK4 are shown.

Although much of the early thinking in the pseudokinase and pseudoenzyme field was that catalytically dead enzymes were likely to function as decoys or substrate traps to modulate signaling flux, there are remarkably few examples of such roles. As observed for other pseudoenzymes (26), it is plausible that pseudokinases could function to sequester substrates away from conventional kinases, although no examples have been reported. Instead, the primary mode of competition mediated by pseudokinases is in the disruption of protein–protein interactions to attenuate signaling flux. For example, a viral ortholog of MLKL was identified to bind to cellular RIPK3 to prevent its engagement and phosphorylation of the cellular MLKL (93). Intriguingly, viral MLKL has evolved a contracted activation loop sequence relative to its cellular cousins, which precludes its phosphorylation by, and disengagement from, RIPK3 (Fig. 6*A*). A similar principle of competitive inhibition has been proposed for the IRAK3 pseudokinase in its role as a regulator of inflammatory signaling *via* the myddosome. The myddosome is a high molecular signaling hub nucleated by the adaptor, MyD88, bringing together the IRAK1 and IRAK4 effector kinases and their regulatory pseudokinase cousins, IRAK2 and IRAK3, to control inflammatory signaling downstream of Toll-like receptor activation (94). IRAK3 was recently identified to adopt a head-to-head homodimer, which was proposed to bind antiparallel IRAK4 kinase dimers *via* C-lobe mediated interactions (39). Such an association is proposed to occlude the IRAK4 active sites, which in turn provides a plausible mechanism for how IRAK3 might negatively regulate IRAK4 kinase activity to attenuate myddosome inflammatory signaling (Fig. 6*B*). These two examples illustrate how protein–protein interactions mediated by pseudokinases and, by extension protein kinases, can occlude substrate access or dampen catalytic activity to attenuate signaling flux.

## Small-molecule modulation of noncatalytic kinase function

Kinases are renowned as a druggable protein class, with more than 50 inhibitors approved by the FDA for clinical interventions. The plasticity of the protein kinase active site makes it malleable to small molecules and provides opportunities for allosteric modulation of surrounding structural features. Thus, small-molecule inhibitors offer the opportunity to block conventional phosphoryl-transfer activity by displacing ATP. Endeavors to target noncatalytic kinase functions with small molecules have been comprehensively reviewed recently (7, 95), with examples now known of modulation of pseudokinase allosteric and molecular switch functions and promotion of competitor behavior. Here, we focus on nascent examples of targeting noncatalytic functions, the technical challenges associated with devising such interventions, and the potential for targeting underexplored pseudokinase/kinase functions. While known examples are dominated by molecules targeting ATP-binding sites in pseudokinases, it is foreseeable that modulating the assembly of signaling hubs might likewise be achieved by compounds that perturb protein interactions. Indeed, the evolution of unique sites that mediate protein–protein interactions outside the pseudoactive site might offer greater prospects for compound selectivity than ATP-binding sites. For example, in TRIB1, the αC helix serves as a ledge for binding to the C-terminal COP1 binding peptide in *cis* (75, 78); displacement of the COP1 binding motif could be achieved through a mimetic small molecule. Not only does the modulation of allosteric, switch, and competitor functions support pseudokinases as plausible therapeutic targets, but also expands the repertoire of activities that could be targeted among conventional protein kinases.

An inherent challenge to developing small-molecule compounds against noncatalytic kinase features is diversity of function, compounding a lack of straightforward enzymatic assay of activity. Accordingly, binding, rather than activity assays—especially differential-scanning fluorimetry (DSF)—has been employed to screen libraries of small molecules to identify pseudokinase binders, borrowing from a system established to characterize nucleotide binding by pseudokinases (9, 43, 96). Prospects for drugging pseudokinases have been thoroughly reviewed relatively recently (7), but, as discussed below, already new examples demonstrate continued development in the area.

The allosteric functions of pseudokinases have emerged as the predominant target for small-molecule interventions to date. Much interest has been shown in modulating the pseudokinase (JH2) domains of TYK2 and JAK2 to control their role as suppressors of the catalytic activity of the adjacent tyrosine kinase domain in each protein (97, 98, 99). Given its involvement in EGFR family signaling and cancer, HER3 has also emerged as a desirable pseudokinase target of therapeutic interest. The formation of an active dimer with HER2 whereby HER3 allosterically activates HER2 is inherent to its function. Stemming from DSF-based screening, a small-molecule binder of HER3 (AC3573) with marked selectivity over HER2 has been identified (100). Importantly, AC3573 antagonizes signaling by decreasing heterodimer formation, in contrast with another ATP-competitive inhibitor, bosutinib, which can promote active dimers (101). The structural basis for the opposing mechanisms of bosutinib and AC3573 is currently unclear, but the contrasting outcomes illustrate that quite divergent allosteric effects are possible. Structural insight will be crucial to further promoting and tuning these allosteric effects. Other receptor tyrosine kinase-like pseudokinases have similarly attracted interest as oncogenic therapeutic targets, where small molecule binding to their pseudoactive sites was proposed as a strategy to regulate interaction with their binding partners. Like for AC3573 binding to HER3, small-molecule binders of ROR1 pseudokinase were identified by DSF and validated as pseudoactive site binders using hydrogen-deuterium exchange–mass spectrometry (HDX-MS) (102). Collectively these examples support the idea that, like kinases, pseudokinases are dynamic in the wild, and their functions can be tuned by interactions with other proteins and small molecules.

Related to modulation of allosteric functions, pseudoactive sites can be targeted with small molecules to control molecular switch functions by locking their conformation to dictate their protein interactions. Such a role was proposed for the MLKL pseudokinase, whose conformational interconversion is promoted by phosphorylation. A small molecule series identified by DSF is known to bind MLKL’s pseudoactive site, although deducing the precise contribution of ligation to pathway inhibition is confounded by the compounds showing affinity for the upstream kinases in the pathway RIPK1 and RIPK3 (54, 58). Similar to JAK2 pseudokinase and kinase domain ligands (103), this illustrates the challenges of pharmacologically targeting pseudokinases selectively, considering their ancestral origins commonly reside in gene or domain duplication events. Additionally, this raises the prospect more broadly that kinase inhibitors may exhibit off-target binding to pseudokinases, which may contribute to efficacy but have gone undetected.

While scaffolding roles of kinases may present a challenge for modulation by small molecules, some examples do exist. For instance, the switchable scaffolding of Tribbles pseudokinases (Fig. 5*B*) appears to be susceptible to modulation by small molecules in the case of TRIB2. Repurposed ligands that covalently modify active site cysteine residues of TRIB2 destabilize the pseudokinase in cells, consistent with a role in activating the scaffold and promoting binding to the COP1 ubiquitin ligase (104). Even where allosteric control of a scaffold is not possible, identification of specific small molecules may serve as tools to define noncatalytic scaffolding activity in cells. For example, DSF and orthogonal approaches—immunoprecipitation *via* ADP-biotin, and fluorescent binding of the nucleotide analog mant-ADP—have enabled identification of ATP-competitive ULK4 inhibitors (82). These compounds provide a starting point for future compound development, but will also prove invaluable to deducing the molecular mechanism of ULK4’s involvement in schizophrenia.

**Figure 7:**
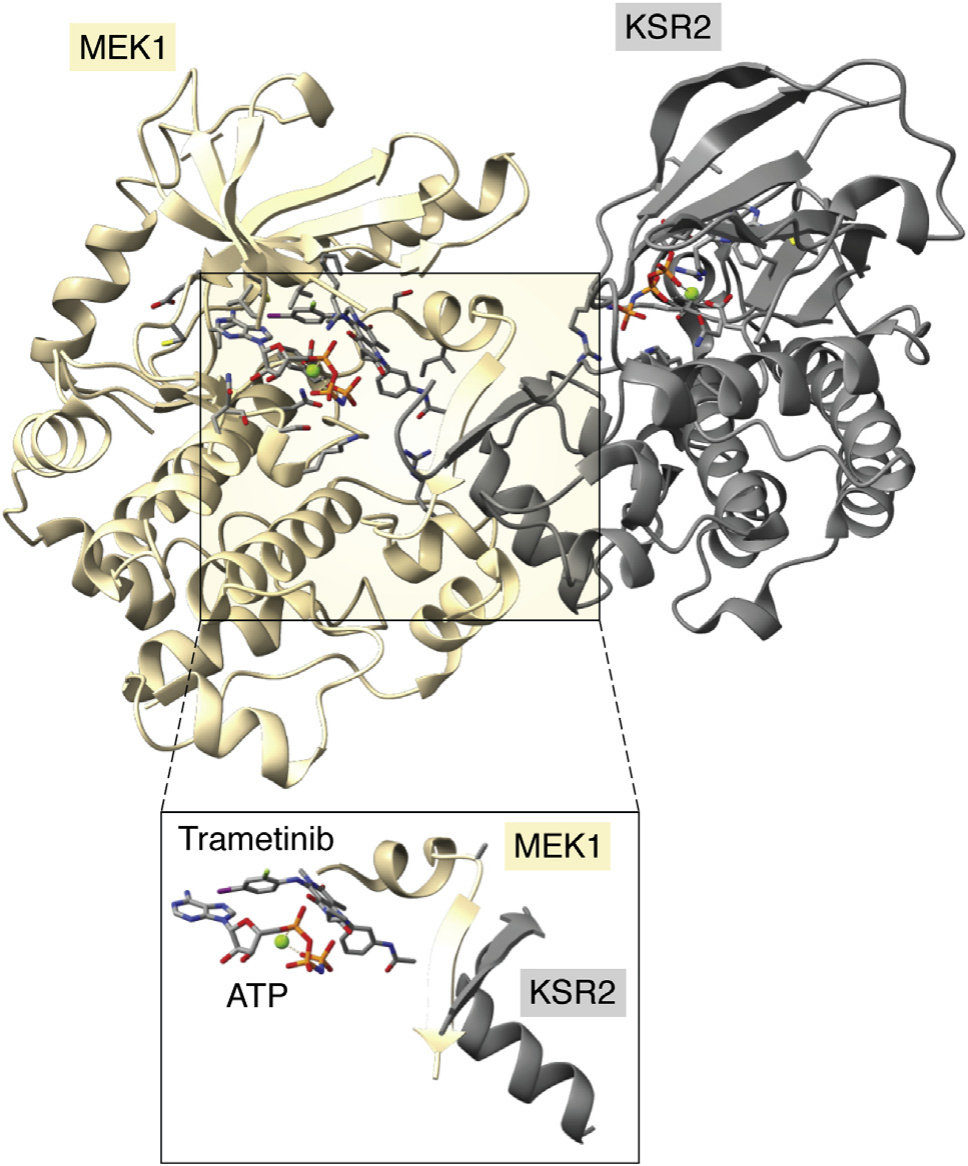
**Structure of the MEK1-KSR2-Trametinib complex.** The overall structure of MEK1 (*tan*) binding to the KSR2 pseudokinase domain (*gray*; PDB 7jur; (107)). An *inset* view below illustrates the intimate relationship between the MEK1 activation loop and Trametinib, which is stabilized by KSR2.

The RAF pathway presents a fresh illustration of how a small molecule can stabilize a competitive pseudokinase complex toward therapeutic ends. The downstream effector of RAF is MEK1, which can form complexes either with RAF kinases prior to activation (105, 106) or with the pseudokinase domain of KSR (63). An elegant recent study demonstrated that the MEK1 inhibitor Trametinib binds in a manner that enhances stability of a KSR-MEK1 complex (Fig. 7; (107)). Not only does this binding mode enhance residency of the inhibitor on MEK1, but the KSR-MEK1 complex is stabilized relative to the RAF-MEK1 complex, diminishing signaling flux through the pathway. In essence, by stabilizing the inhibitory complex between MEK1 and KSR trametinib amplifies the ability of KSR to compete with RAF-MEK1 complexes. Trametinib was first identified by phenotypic screening, but approved by the FDA in 2013 as an agent to antagonize MEK1 in metastatic melanoma. Extensive structural information allowed the authors to specifically design a compound with enhanced ability to stabilize KSR-MEK1, which shows promising signs in terms of avoiding adaptive resistance to MEK1 targeted therapy. The KSR-RAF-MEK1 signaling axis provides a template for possible intervention among other pseudokinases. For instance, small molecules may prove useful in regulating IRAK3 pseudokinase conformation such that IRAK3 engagement of the conventional kinase, IRAK4, is stabilized (Fig. 6*B*; (39)), and errant inflammatory signaling can be dampened therapeutically.

It is foreseeable that competitor functions could be mimicked by small molecules more broadly, although identification of compounds that disrupt protein–protein interactions looms as the major challenge in that pursuit. For example, compounds that occlude MLKL pseudokinase binding by RIPK3 kinase—emulating the function of viral MLKL—are plausible necroptosis pathway inhibitors, but may prove challenging to identify. One prospect for discovery of such inhibitors is virtual screening, as recently illustrated in the identification and validation of STRADα pseudoactive site binders (108). Alternatively fragment screening may be appropriate, which has been widely used for conventional kinases but, to our knowledge, not yet reported for pseudokinase studies.

The examples outlined here employ a combination of methods including DSF, immunoprecipitation, fluorescence-based assays, virtual-screening, HDX-MS, and structural analysis. Most importantly, they all rely on specific knowledge of noncatalytic kinase function built over years of fundamental research. These manifold efforts have already demonstrated utility, or potential, for modulating the various classes of noncatalytic kinase function: allostery, switching, scaffolding, and competition. Only through detailed mechanistic studies has it been possible to attribute these noncatalytic functions to kinases and pseudokinases in cell signaling. As our fundamental understanding of noncatalytic kinase functions in cell signaling continues to grow, we expect further, currently unexplored avenues for pharmacological intervention to emerge.

## Conflict of interest

J. M. M. is engaged in developing necroptosis inhibitors in collaboration with Anaxis Pharma Pty Ltd. P. D. M. declares no conflicts of interest with the contents of this article.
